# Using Virtual Reality Social Environments to Promote Outcomes' Generalization of AVATAR Therapy for Distressing Voices: A Case Study

**DOI:** 10.1002/jclp.23785

**Published:** 2025-03-18

**Authors:** Mar Rus‐Calafell, Nils Ehrbar, Tobias Teismann, Silvia Schneider, Ekincan Tas, Simon Schuster, Clementine Edwards, Mark Huckvale, Thomas Craig, Philippa Garety, Thomas Ward

**Affiliations:** ^1^ Mental Health Research and Treatment Center, Faculty of Psychology Ruhr‐Universität Bochum Bochum Germany; ^2^ German Center of Mental Health (DZPG), Partner Site Bochum/Marburg Bochum Germany; ^3^ Department of Psychology, Institute of Psychiatry, Psychology & Neuroscience King's College London London UK; ^4^ Avatar Therapy Ltd London UK; ^5^ Department of Health Service and Population Research, Institute of Psychiatry, Psychology & Neuroscience King's College London London UK

**Keywords:** auditory hallucinations, AVATAR therapy, cognitive models, digital health, relational approach, virtual reality, voices

## Abstract

AVATAR therapy (AT) works by facilitating a ‘face‐to‐face’ dialog between the person and a digital representation (avatar) of their persecutory voice. Although there is cumulative evidence of this way of working with voices, enhancing the therapeutic focus on improved confidence and a sense of control of the voices in social situations represents a promising way to boost the generalization of therapy gains into social contexts. This paper presents a descriptive clinical case example of AVATAR_VRSocial therapy, a new augmented version of AT incorporating immersive Virtual Reality to help the person deal better with their voices in daily situations. “Laura” is a woman who was hearing a very distressing, threatening voice. She felt anxious and distressed when anticipating hearing it and would engage in safety‐seeking behaviors to prevent hearing the voice. Laura was supported to stand up to her avatar and regain power over it by using assertive responses, both in active avatar dialog and when exposed to the avatar voice in VR scenarios, which turned into reduced distress when hearing the voice in her everyday life. Laura's dialog with her avatar evolved into a more explicit exploration of the meaning and the purpose of the voice in relation to previous trauma and personal relationships. The additional work in VR appeared to facilitate exposure to social situations while hearing the distressing voice, without performing seeking‐safety behaviors, and to allow for practicing strategies to reduce the voice's interference, which evolved from the dialogic sessions with the personalized avatar.

## 1 Introduction

AVATAR therapy is an innovative therapy approach designed to support voice hearers in developing an increased sense of power and control over their voices, through a series of dialogs between the person and a digital representation of the voice (or avatar). It was originally created by Julian Leff in 2013, in the United Kingdom. AVATAR therapy involves direct exposure to the anxiety‐provoking fear stimuli (i.e., the representation of the voice and specific distressing content), providing an opportunity for the voice‐hearer to face their voice within a controlled and safe space and take back control over feared and disempowering experiences (Ward et al. 2020). Previous research has also shown that participants' sense of voice presence (the individual's perception of voice embodiment, real‐time communication, and enactment of the relationship) is maintained across therapy sessions and that significant reductions of anxiety during dialog are successfully achieved, especially after the first three therapy sessions (Rus‐Calafell et al. 2020). The continuous exposure to the experience of seeing and hearing the voice‐over therapy sessions, along with the modification of the relationship with the voice, is thought to contribute to the disconfirmation of maladaptive beliefs about the voice and others and, thus, a reduction of the voice's associated threat and distress. Preliminary evidence for its efficacy was demonstrated through two independent pilot studies (Leff et al. 2013) (Percie du Sert et al. 2018) and further supported by the findings of a large fully powered randomized controlled trial (AVATAR1) comparing AVATAR therapy and Supportive Counseling (Craig et al. 2018). This study demonstrated a large effect size (*d* = 0.8) for AVATAR therapy on the primary outcome (total score on the Psychotic Symptom Rating Scales, auditory hallucinations subscale). There is an ongoing UK‐based multi‐center randomized controlled study collecting new evidence of its efficacy (AVATAR2 trial). In this trial, two versions of the therapy (a brief and extended form) are being tested against treatment as usual, to support optimization. Therapy is being delivered across geographically diverse sites in England and Scotland with a view to supporting wider implementation in the National Health Service (NHS).

Although effects on social functioning were not reported in the AVATAR1 RCT (Craig et al. 2018) due to the lack of suitable validated measures included within the assessment battery, participants who took part in the embedded qualitative study reported an increased level of engagement in social situations and improved confidence around others (Rus‐Calafell et al. 2022). Since voice‐hearers may also present with reduced social functioning due to difficulties in relating their experience of voice‐hearing to others and fear of being negatively perceived in social situations (Sheaves et al. 2021), generalization of therapy gains appears as a clinical priority for those experiencing negative voice‐hearing. Enhancing the therapeutic focus on improved confidence and a sense of control of the voices in social situations represents a promising way to boost the generalization of AVATAR therapy gains into social contexts and reduce anxious avoidance and threat cognitions (including perceived threats from the voices).

Virtual reality (VR) has long been used to facilitate exposure to feared stimuli, mostly for anxiety disorders, with encouraging results for the treatment of psychosis (Bell et al. 2020; Rus‐Calafell et al. 2018). Virtual environments evoke responses in a participant that match those occurring in the natural environment. This offers a unique opportunity to access the user's real‐time behavior, including interaction with virtual agents, and allows the person to test out new responses, for example, targeting the safety behaviors and paranoid attributions that can maintain voice‐related distress (Freeman et al. 2022; Pot‐Kolder et al. 2018). Relevant environments, providing emotionally significant content targeting specific emotions and related cognition for a specific population, and optimal induced levels of presence are required when delivering virtual reality‐based therapy. Following this evidence, we present a new augmented version of AVATAR therapy, which includes three additional sessions using VR environments to improve the generalization of therapy gains to the person's social functioning.

### 1.1 AVATAR Therapy and VR Social Environments

AVATAR therapy works by facilitating a novel therapeutic context in which a ‘face‐to‐face’ dialog is facilitated by a therapist between the person and a computerized representation of their voice. Bespoke software transforms the therapist's voice to match the pitch and tone of the chosen voice, and the person creates a visual representation of their voice. The voice and image are combined to produce the ‘avatar’ (i.e., an animated talking virtual agent with enhanced lip‐sync speech and eye blinking), through which the therapist interacts with the voice‐hearer, in a three‐way dialog (for further details on the software, please see https://www.avatartherapy.co.uk/). Using this platform, the therapist can speak to the client in their normal voice or in the chosen avatar voice, which is a modified version of their normal voice. Where there are multiple voices, the participant selects one to work with (usually the most dominant, frequent, and distressing). The embodiment of the voice is enhanced using direct verbatim speech and enactment of the ascribed character and background of the voice. A comprehensive account of therapeutic targets of AVATAR therapy showed that some therapeutic targets were clearly present for all therapy completers (power and control, self‐esteem, and future focus), whereas others (e.g., maintenance processes, working with trauma, experiential disengagement) were identified in some but not all participants, in line with the tailoring of the intervention to an individualized formulation (Ward et al. 2020).

**Figure 1:**
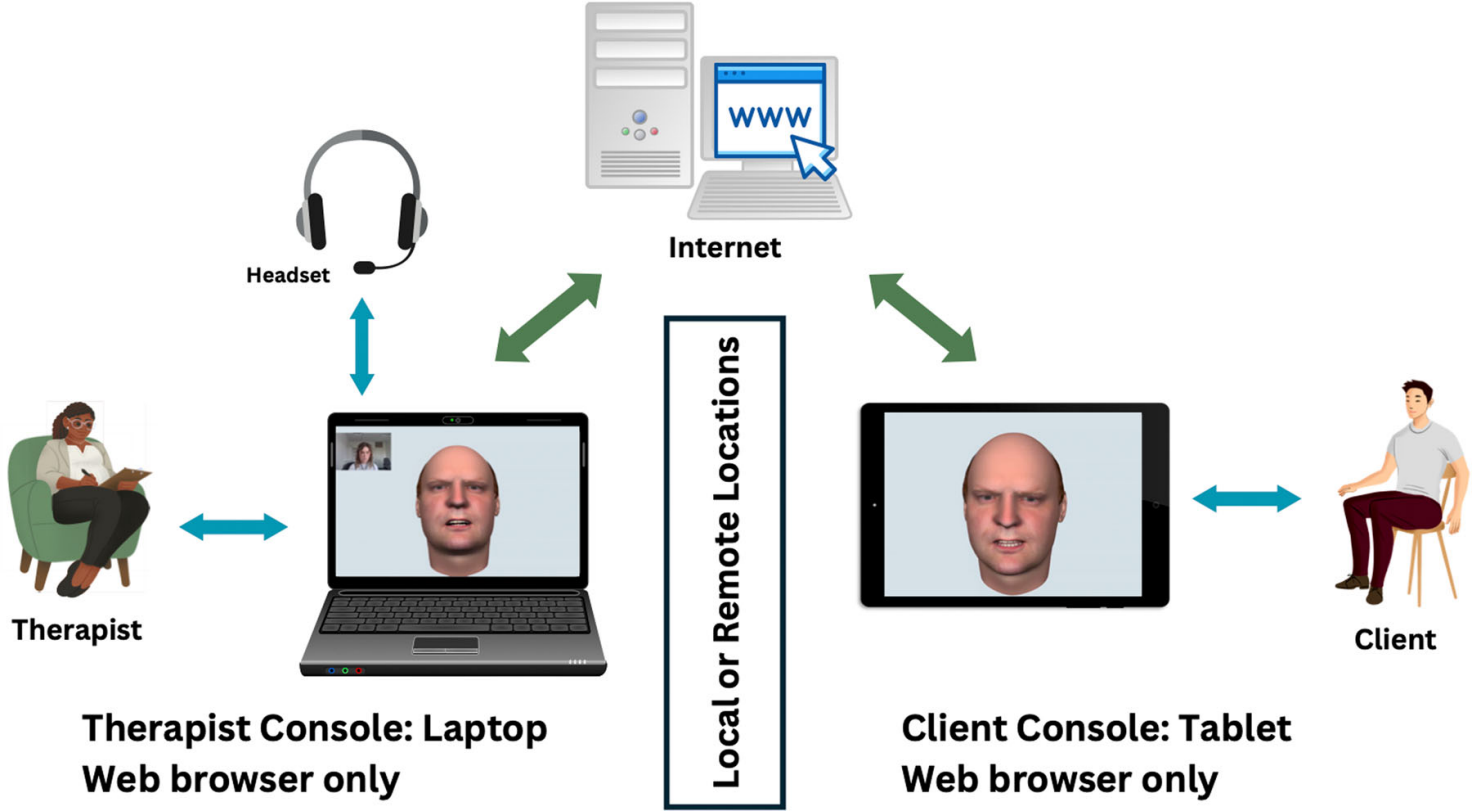
Set up for AVATAR therapy.

AVATAR therapy, as delivered within the pilot work and AVATAR1 trial, consists of a total of 6 sessions (in addition to an initial clinical assessment session including avatar creation) and comprises two phases. In the first phase (Exposure and Assertiveness), the avatar delivers verbatim voice content (including threats and abuse), and the person practices assertive responding. Over time, the avatar becomes less hostile as the person develops increased power and control within the dialog. This cues a second phase with formulation‐driven therapeutic targets, which can include work on beliefs about voices, self‐concept, and trauma. Each AVATAR therapy session consists of three parts: pre‐dialog, Active dialog, and post‐dialog debrief. The pre‐ and post‐dialog are important for cueing and consolidating key change processes occurring in the dialog with the avatar. The active dialog duration is approximately 5 min in early sessions, increasing to 10–15 min in later ones. Participants and therapists are in separate rooms, but in constant communication, for this part of the session, as shown in Figure 1.

**Figure 2:**
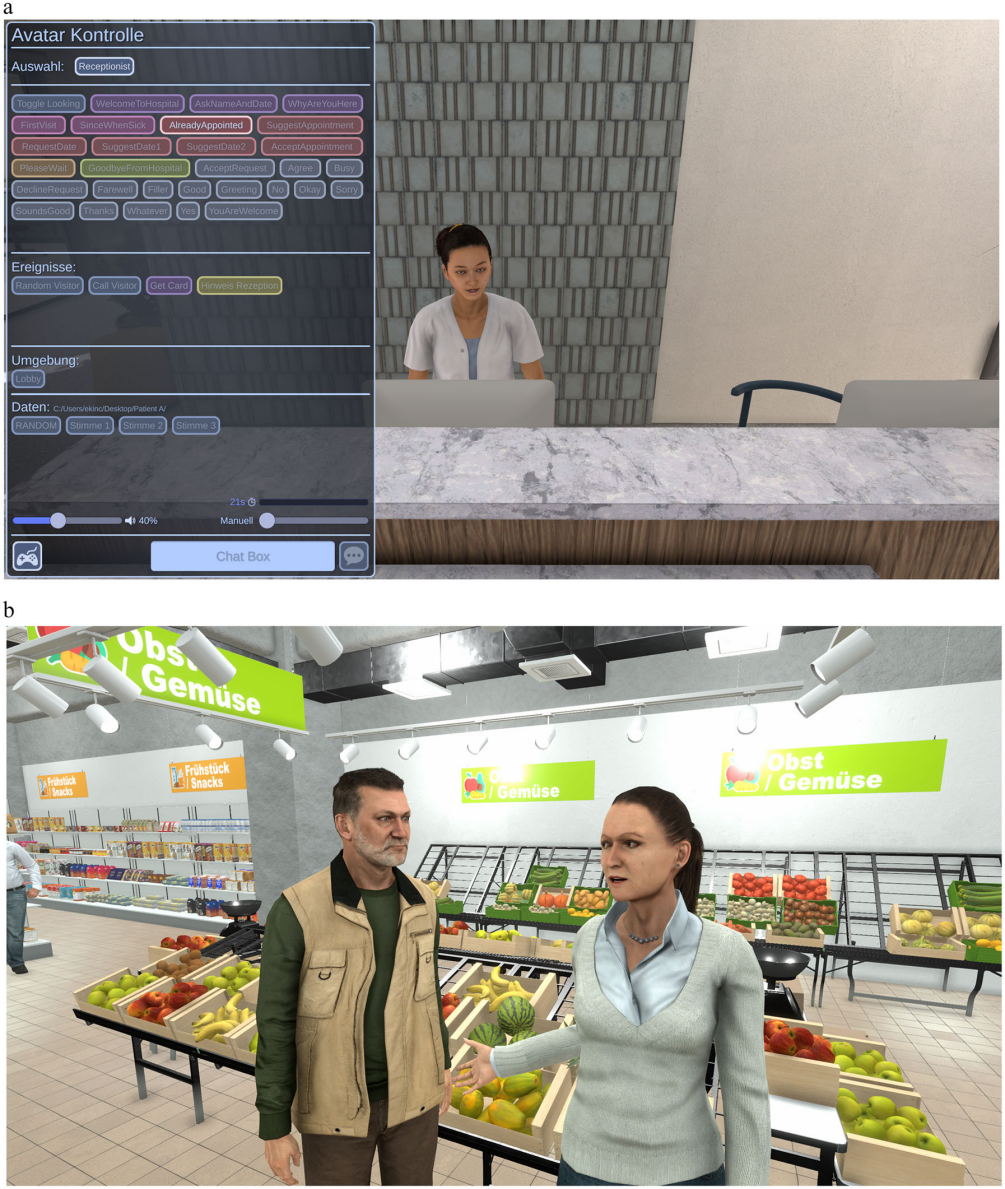
(a, b) Screen captures of the VR scenarios and control commands for the therapist.

For the therapy approach presented in this case study, two social virtual scenarios have been created to facilitate the generalization of AVATAR therapy learning into the management of the voice‐hearing experience during social interactions. The two scenarios are (1) a waiting room from a mental health service, in which the person is encouraged to make an appointment at the service desk; (2) a supermarket, in which the person is asked to look for a specific product and ask for help to the members of the staff. Each scenario has, therefore, a specific task to be accomplished by the person. Some responses from the virtual agents are automated and modulated by the person's verbal response (see Figure 2).

The virtual scenarios are presented using a wireless head‐mounted display (Pico 4) with a resolution of 2160 × 2160 pixels per eye, 122.16° diagonal field of view, and mounted headphones. The personalized negative voice is exported from the AVATAR therapy platform to the VR scenarios. The therapist can trigger the negative voice at random intervals (determined by the system) or at his/her own demand depending on the person's performance. As per the AVATAR therapy sessions, the three VR sessions consisted of three parts: the pre‐VR discussion with the therapist, the immersive part using the VR headset (lasting approximately 10 min), and the post‐VR debrief with the therapist. The person can request to stop the immersive experience at any point. The scenarios were developed based on the team's prior clinical and research experience with using VR to address social difficulties in people with psychosis (Rus‐Calafell et al. 2018). Initial proposals were selected and shaped by the recommendations from the Live Experience Advisory Panel (LEAP) at the Clinical Psychology and Digital Psychotherapy Department, Ruhr‐Univerität Bochum, which was established after receiving the grant that supports this study.

The addition of VR scenarios to AVATAR Therapy has resulted in a new augmented version of this digital therapy for voices: the AVATAR_VRSocial Therapy (Table 1).

**Table 1:** Summary of the sessions included in AVATAR_VR Social therapy.

	Short description	Number of sessions
AVATAR therapy	Three‐way dialog between the participant, avatar, and therapist	7 sessions (6 sessions + 1 assessment and avatar creation session)
VR social scenarios	Interaction with social agents during quotidian situations + personalized negative voice	3 sessions

## 2 Case Illustration

### 2.1 Presenting Problem and Client Description

Laura is in her mid‐thirties and has a diagnosis of non‐affective psychosis. She was referred to our outpatient clinic (Mental Health Research and Treatment Center, MHRTC, Ruhr‐Universität Bochum) by her clinical psychologist, who met Laura during her last in‐patient admission. Laura was experiencing highly distressing auditory verbal hallucinations at the time of referral.

Laura identified herself as a German person and added that her mum had an Indian background. In her own recollection, she started hearing voices at the age of 13. At that time, Laura was living in a foster home as a result of a compulsory measure by child protection services in her home state. During the clinical assessment, she explained that her parents separated very early in her life and that her mother was suffering from mental health breakdowns, though she did not know whether she ever got a formal mental health diagnosis. Before living in the foster home, she was under her mother's care only. The core family consisted of her mother, her two sisters, and herself. Laura explained that she went through challenging experiences, such as vicarious trauma of exposure to physical and verbal abuse perpetrated by her mother to her sisters, as well as direct experience of emotional neglect. While in the foster home, she began to hear a child's voice crying and calling out to her. When Laura shared this experience with her carers at that time, she felt dismissed and highly stigmatized. She described how this experience made her more reserved about sharing her struggle with negative voices with others, including health professionals.

Laura was first in contact with mental health services at the age of 15. Over the course of her life, she heard mainly one voice, sometimes two, with some other voices in the background (without clear content). Her voice hearing shifted in content and personification, and during episodes of acute psychosis, she often felt the voice was more positive than during the periods she was in remission. Four years before AVATAR therapy, she started hearing the voice of Conrad, an abusive voice attributed to an extra‐terrestrial humanoid being, that constantly threatened her with violence (saying things like “*I will kill you*”). At the time of starting therapy with our team, Laura was engaged in professional training and she was able to attend her classes and exams. However, although she had her own flat, she would spend most of her free time and nights over at her partner's. She would refuse to undergo social activities alone or remain alone in her flat, as she feared that she could hear the voice and “become unwell”. Although she described her interpersonal relationships as “satisfactory”, she was debating whether her current friends were really supportive. She refrained from talking about her partner during the clinical assessment, but details about their relationship emerged during the AVATAR therapy sessions (see section 2.4).

### 2.2 Therapist

The therapist working with Laura was a male psychotherapist in his mid‐thirties with 3 years of experience working with people with psychosis. He studied Psychology (BSc) and Clinical Psychology (MSc), before enrolling into his clinical training. His main approach as per training is Cognitive Behavioral Therapy (CBT). In Germany, training as a licensed psychotherapist takes at least 3 years to complete, including a final mandatory state examination. The training includes 600 h of theoretical training in the chosen therapy approach (CBT, Psychoanalysis, System Therapy, or Depth Psychology) and a minimum of 2400 h of supervised practice (including inpatient and outpatient settings).

### 2.3 Case Formulation

Laura explained that she was hearing one distressing voice (Conrad) several times a week for several minutes, and its loudness could fluctuate over time. The experience was mainly triggered when she was alone in her flat, but it could also occur when she felt upset, for example, in the company of her partner. She explained that she would feel highly anxious and distressed when anticipating hearing the voice threatening her and would avoid being alone. Therefore, she would carefully plan her days in a way that she would constantly be around known others or at her partner's flat, to avoid hearing the voice. On those occasions when she was alone, she would call friends or acquaintances by phone, to prevent hearing Conrad. She described feeling constantly afraid of Conrad appearing and threatening her.

Laura felt that “if her mother had shown her more love, she might have had a more positive prospect of her life and would be able to show more love towards herself”. Through her life experiences, Laura appeared to have developed a very negative self‐concept, characterized by a view of herself as a “negative influence for others*”* and a constant burden to those who loved her (as she was “too needy, and dependant”). This view of not being worth the care and attention of others also seemed to be reflected in her relationship with her voice and her strong belief of deserving its constant threat and mistreatment. The basis for this belief has been a recurrent feeling of being a burden to others, especially romantic partners. Laura's interactional style of being fixated on close personal relations is present in the context of angry and abusive reactions from others, and thus, for Laura, led to a feeling of being a negative part of these others' lives.

She perceived her voice as very powerful while also feeling unable to speak up to it in fear of retaliatory actions (ultimately, harming her physically). Although she perceived the relationship with Conrad more positively in the past, their relationship had deteriorated during the last months, with Conrad becoming increasingly abusive. The formulation suggested potential links between feelings of vulnerability and powerlessness around Conrad and early experiences of childhood trauma. In terms of social interference of the voice, Laura explained that she would use interactions with others as a form of avoidance to hear the voice. She would regularly talk to friends and her partner on the phone when she was somewhere alone and feeling that the voice would start.

### 2.4 Course of the Treatment

As explained above, the therapy was planned as follows: six sessions of AVATAR therapy (with an additional assessment and avatar creation session, as described by Craig et al. 2018) and three sessions of VR‐based training to practice managing interference of distressing voices while in social situations (with unknown others).

During the assessment session, both the therapist and Laura discussed her experience of voice hearing, including specific verbatim content. This session also included the creation of the avatar (i.e., tailoring the image and sound of the avatar to match the voice). Laura showed a strong and fearful response to the avatar, even though she reported having no clear visual image of him beforehand. She described it in a way as making him more “real” by giving him a visual representation.

During the early therapy sessions (1 and 2), Laura appeared very reserved during the pre‐dialog part, which the therapist understood to be linked to her prior experiences with mental health professionals. However, during these early dialogs with the avatar, she would disclose some relevant details about her personal life or relationship with Conrad that she didn't previously share with the therapist. This posed a challenge for the therapist, as he had to improvise his responses as the avatar, and additions to the dialog had to be incorporated spontaneously while also trying to retain a sense of voice‐presence and reliable enactment of the relationship. In line with the standard AVATAR therapy manual and targets (Ward et al. 2020), the focus of the dialog with the avatar was on asserting power and control. Laura was encouraged and supported by her therapist to stand up and challenge the avatar's threats. This new “sharing space” between Laura and her avatar opened the door for a transitional phase and a more compassionate attitude from the avatar by acknowledging her strength, commitment, and trust in him. During the dialogs, Conrad and Laura also discussed some maintenance processes of her voice‐hearing experience: why he would appear when she was alone or how worrying and avoiding hearing him was also allowing him to gain more control over her.

After session three, the therapist had to support Laura with what appeared to be an emotional crisis after an argument with her partner. It was after this crisis that Laura also opened up about experiencing physical and verbal abuse from her partner, as well as verbal abuse by his family (for which she was offered additional support from the therapist and the possibility to liaise with other services). Laura interpreted this abusive behavior as something “she deserved*”* while also appreciating the resemblance to her relationship with Conrad. Ultimately, she felt very dependent on her partner, also as an escape from her distressing voice‐hearing experience. While exploring this further, she reported having similar abusive relationships and dependency issues with previous long‐term romantic relationships. This emotional crisis was used to illustrate how her relationship with Conrad could potentially be mirroring her other personal relationships in which she would typically assume a submissive relating style.

During phase 2 (sessions 4–6), Laura continued to consolidate her learning on gaining control and power over her avatar by dialoguing with him about boundaries, respect, and supportive behavior (at her request). Although she seemed to accept the avatar's willingness to become more supportive and helpful, she remained skeptical about how much she could trust him. An abbreviated outline of the dialog mid‐therapy course between Laura, Conrad, and the therapist is given in Table 2.

**Table 2:** Adapted transcript of the dialog between Laura and Conrad in the third therapy session (original dialog is in German).

Session 3	AV: How are you?
	Pt: I'm okay, how are you?
	AV: What do you think?
	Pt: Too good, right?
	AV: You think I'm feeling well?
	Pt: Yes.
	AV: Why do you think that?
	Pt: [quietly] Because you scare me… [now louder] you really scare me.
	AV: Do you think this is fun for me?
	Pt: I think so.
	Tx: Laura, how are you now?
	Pt: It's intense. Makes me shake a little.
	Tx: I think it's great how you instantly enter the dialog and respond to him. You present very brave.
	Pt: When N (the therapist) is not with me anymore, how will we get along? I will fear you then.
	AV: I've got a feeling; you do not have to fear me anymore. [avatar conceding]
	Pt: I really hope it won't come this way [referring to “being scared”].
	[pause]
	AV: You are talking to me in a completely different manner already now.
	Pt: Because I am afraid you might do something bad to me.
	AV: I am scared sometimes, too. But you are defending yourself against me now.
	[pause]
	Pt: It's good when I don't hear you, or even when you are there but I cannot understand you.
	AV: That's almost mean.
	Pt: Yes, maybe for you. Not for me. It's better for me, when you don't scare me.
	[pause]
	Tx: Laura, today you've really got it going on. You are really giving him a piece of your mind today!
	AV: I can see you are not the pushover I thought you were. [avatar conceding]
	Pt: Once N (the therapist) is not with me anymore, I must be able to deal with you on my own. I'll be strong.

Abbreviations*:* AV, avatar; Pt, patient; Tx, Therapist.

A more compassionate positioning in their relationship from the avatar in sessions 4 and 5 facilitated a shift to a more future‐oriented positive outlook on their relationship. The avatar also acknowledged that, in the past, he had threatened and dismissed her because he did not see her as strong enough to stand up for herself, but that this was changing. Although the therapist introduced the idea of asking a loved one to create a list of positive qualities about Laura, she chose not to engage in this aspect of the protocol (even when the therapist suggested that he could provide one if Laura did not wish to ask a loved one). It should be noted that work targeting self‐esteem can be understandably challenging where entrenched negative schema is present. In such instances, the AVATAR Therapy manual identifies the need to target self‐concept over a longer period of time both inside and outside of a dialog, formulating the person's aversive response to “positive qualities”. By the end of the AVATAR therapy sessions, Laura and the avatar came to an understanding of her being in charge of the relationship and their interactions. This was further facilitated by positive experiences at her training and workplace, as well as potential new social relationships with colleagues who seemed to Laura more trustworthy. Laura expressed to the therapist that she got a feeling of “being more in control of her life*”*.

As part of the planned VR augmentation, three therapy sessions using VR environments followed AVATAR therapy sessions. The objective of these sessions was to help Laura feel more confident to be around others and in social situations, without the need to immediately run to her partner's flat or call someone to prevent her distressing voice hearing (seeking safety behaviors). The most distressing verbatim content uttered by Conrad *(“I will kill you”; “I will use your body”*) was prerecorded using the AVATAR platform and exported to the VR scenarios. This verbatim could be triggered at random intervals or by the therapist at any point during the virtual interactions.

In the first session, Laura was immersed in a virtual supermarket where she had to find certain foods and request help from others to do so. During this, she exhibited avoidance of social interaction while hearing her negative voice (and she walked around the environment without any sort of interaction despite the instructions given). This was modified in the following sessions with her testing out various strategies during social interaction: to ask the voice to stay silent and to re‐focus her attention on the planned task and conversations with the virtual agents. Laura managed to achieve this with encouragement from her therapist. Specifically, the strategies she used were standing up to the voice, explicitly asking her voice to not hinder her “success”, and reminding Conrad of the agreement they had in place (she would decide when it was helpful for her to interact with him). In these instances, the therapist would purposely reduce the frequency of the voice‐hearing during the virtual encounters, to incorporate positive reinforcement to Laura's behavior of managing the voice hearing. Laura managed to complete successfully all the environments with their respective tasks.

### 2.5 Outcome and Prognosis

Laura completed the whole therapy course and attended a final clinical assessment after 14 weeks. As part of this assessment, several clinical measures were used to evaluate her progress: Psychotic Symptom Rating Scales, auditory hallucinations subscale (PSYRATS–AH, Haddock et al. 1999), and the Oxford Cognition and Defences Questionnaire (O‐CDQ, Rosebrock et al. 2022). Outcomes based on the PSYRATS showed a decrease in the total burden of voice‐hearing to almost half of the score reported before therapy, as well as a reduction in frequency and associated distress dimensions (> 5 units, based on the dimension calculation proposed by Woodward et al. 2014). In terms of anxious avoidance, there was a reduction in the three subscales included in the O‐CDQ: threat cognitions, anxious avoidance, and safety behaviors.

The clinical impression from the therapist was that while the main therapeutic change was on Laura's perceived sense of control of her voice, important functioning and independence changes were achieved, such as her personal goal of sleeping at her own place most of the nights (as opposed to staying with her partner), and less reliance on being with known people as well as a positive sense of achievement in relation to her studies and social life. Specifically, her outlook on social relations has improved and Laura wants to spend more time with colleagues and other students at her school, who she feels won't take advantage of her. Furthermore, she reported a distinct shift in her outlook and was more focused on her future and how she might shape it positively. Although Laura remained hesitant to share her experience of voice‐hearing with others, she expressed her desire to become part of the Lived Experience Advisory Panel for the subsequent AVATAR_VRSocial Therapy feasibility clinical trial conducted by our team.

Adverse events (AEs) were monitored during the course of the therapy, following the same criteria used in AVATAR1 and 2 clinical trials (Craig et al. 2018, Garety et al. 2021). AEs are defined as any untoward medical occurrence, unintended disease or injury, or untoward clinical signs in service users/patient participants, whether or not related to the therapy or device, which require additional support or input from health professionals. Adverse events will be initially assessed at three levels of intensity: mild, moderate, and severe, which reflect the impact of the event on the person at the time. Please note that there is a distinction between ‘severe’ and ‘serious’. Seriousness is the criteria for defining regulatory reporting obligations and the following will be considered as serious adverse events (SAEs, categories A–C): All deaths (category A), incidents that acutely jeopardize the health or psychological wellbeing of the individual, resulting in immediate hospital admission and/or permanent disability (category B) or resulting in injury requiring immediate medical attention (category C). Consistent with the findings of the large‐scale clinical trial by Craig et al. (2018), where no therapy‐related SAEs were found, there were no SAEs during the delivery of AVATAR_VRSocial with Laura. However, Laura unfortunately lost her father during the course of the therapy, with whom she had sporadic contact. The therapist managed this event closely by providing multiple supportive phone calls, and there was no perceivable impact on the therapy (except for an agreed 2‐week break while dealing with the funeral arrangements).

### 2.6 Personal Experience

Laura consented to a feedback interview with a member of the clinical team after completing her therapy course. The interview took place at the MHRTC and lasted around 1 h. During the interview, Laura appeared relaxed and willing to share her experience with the therapy and her therapeutic relationship with her therapist. Laura first learned about the therapy from her psychotherapist during her admission at a local mental health facility. When asked about her initial thoughts about taking part, Laura recalled neither feeling afraid nor apprehensive about the prospect of the therapy; instead, she had been intrigued and simply asked herself, “why not?*”*.

Despite this, she described the actual creation of the avatar as an “intense experience” for her. Hearing what she reported as a near‐perfect copy of her distressing voice coming from the speakers for the first time had felt strange and unsettling. When asked to summarize her first exposure to the avatar, Laura paused and simply said, “that was crazy, you know*?*”

However, this first intense contact with the avatar did not stop Laura from continuing with the sessions. One factor that contributed to this was the strong bond she built with the therapist. Reflecting on her experience, Laura frequently highlighted that this relationship had been one of the most important parts of the therapy for her. According to Laura, the main reason for this was her previous struggles with shame and fear when it came to sharing her experience of hearing distressing voices. In the past, these feelings had prevented her from opening up completely about her experience, even to other therapists. This time, however, due to feeling a genuine sense of interest and understanding from the therapist, Laura found herself able to speak freely about the voice to a degree she had not done before: an experience she described as “beautiful”. The resulting bond of trust also helped her overcome her fear and stand up to the avatar frequently during therapy sessions.

Nonetheless, challenging the avatar remained difficult for her throughout the treatment, especially during the immersive VR sessions. Laura recalls one particularly intense VR session where she pleaded with the therapist to “stay with her” because she felt almost overwhelmed with fear. However, this did not make her want to avoid or stop the VR sessions. Partly this was because the stress never lasted beyond the VR part itself, as she could always reassure herself afterward that it was “just a simulation”. Indeed, Laura also emphasized how *“*cool*”* it was to walk around in virtual environments and interact with the digital characters, and she ultimately hoped that the technology would develop even further.

When asked directly about changes in her voice‐hearing experience, she said that she could still hear Conrad. Her fear of confronting him remained “strong”. However, Laura was able to challenge him and was not discouraged by the fact of still hearing some threats from him. She said that she “simply needed more time to work on herself*”*. Laura also highlighted how important the therapy had been to her to understand her experience of hearing voices, how it had improved her sense of self‐worth; and how it had allowed her to speak freely about her experiences to a degree that she had not felt able to do before. Laura ended the interview with the wish that more therapists would be trained in the “treatment of voice‐hearing”, so that people like her could get the help they needed.

## 3 Clinical Practices and Summary

This clinical study presents, for the first time, the clinical application of the integration of an immersive VR module to AVATAR therapy (as delivered by Craig et al. 2018), with the aim to generalize therapy learnings to the management of voices during everyday social life. As shown in previous studies (see e.g. (O'Brien et al. 2021; Rus‐Calafell et al. 2022), AVATAR therapy enables voice‐hearers to engage in face‐to‐face dialog with a carefully characterized digital representation of their persecutory voice. The primary focus of this dialog during early sessions is for the person to gain power, control, and autonomy. Based on the individual's clinical formulation, subsequent dialogs develop incorporating autobiographical context, meaning‐making, and experiences of trauma and powerlessness (Steel et al. 2019) (Hardy et al. 2024). During the new three additional VR sessions, the person is empowered to manage their voices better when they are in social situations and interacting with others, by applying some of the learning acquired during AVATAR sessions.

With respect to wider clinical implications, it should be noted that, as with the standard AVATAR therapy, the delivery of AVATAR_VRSocial requires updated knowledge of cognitive and relational models of auditory verbal hallucinations in psychosis, sensitivity, and effective training and supervision. Additionally, psychotherapists must be skilled in the use of technology during psychological treatment. AVATAR therapy presents several delivery challenges, such as the need for therapists to switch between speaking as themselves and as the avatar in real time. It also raises ethical concerns, particularly when therapists' voice abuse through the avatar, reenacting critical or harmful relationships. Clinicians must be aware of the impact of this approach and sensitive to emotionally loaded content, especially related to abuse and discrimination. Working with verbatim content can offer a powerful opportunity for voice‐hearers to reclaim control over words that have previously silenced or disempowered them. It is crucial to assess and regularly review the voice‐hearer's understanding, support system, and readiness for this approach. The therapeutic relationship is central, with a focus on respect and collaboration. It is important to consider how past experiences may influence the relationship, particularly in supervision, where attention should be given to the dynamics of power (Ward et al. 2020). In a recent qualitative study (Rus‐Calafell et al. 2022), all interviewed participants who underwent AVATAR therapy reported positive engagement with their therapists and felt well‐supported. They emphasized that both the consistent dialog and personalized positive feedback helped strengthen the therapeutic alliance, encouraging them to try ‘new things’ within a safe environment. Additionally, it was found that a strong therapeutic alliance was maintained despite the active dialog being delivered through a digital platform, with the participant and therapist located in separate rooms.

All the AVATAR therapy to date, delivered as part of the present study, by Garety et al. (2021), Craig et al. (2018) and Leff et al. (2013), has been delivered by clinicians who underwent clinical training and regular peer supervision, with the main clinical overseeing the different clinical research studies. Training involves a combination of direct teaching and self‐directed learning (including access to live treatment reference material), followed by closely supervised training cases. Sharing of live audio is a key aspect of supervision to inform discussions around the key treatment processes to be targeted (both in terms of enacting the avatar and suggested consolidation work before and after dialog).

To summarize, Laura was supported to stand up to her avatar and regain power over it by using assertive responses, both in active avatar dialog and when exposed to the avatar voice in VR scenarios, which turned into reduced distress when hearing the voice in her everyday life. Furthermore, Laura's dialog with her avatar evolved into a more explicit exploration of the meaning and the purpose of the voice in relation to Laura's previous traumatic experiences and personal relationships. Interactions were used to process these past experiences and to help develop a more positive sense of self‐respect, as well as a more positive view of the future. The additional work in VR appeared to elicit even higher levels of anxiety and arousal than the dialogic sessions. However, it facilitated exposure to social situations while hearing the distressing voice, without performing seeking‐safety behaviors (such as escaping or talking to known others), and to allow for practicing strategies to reduce voice interference. The positive and trustworthy therapeutic relationship with her therapist was identified as key for Laura to engage and complete the therapy. Despite the high levels of arousal during early dialogical sessions and virtual exposure, Laura felt safe and confident to continue with the work together. Although she was still hearing the negative voice at follow‐up, both frequency and associated distress had decreased notably. There was also a decrease in social avoidance and frequency of threat cognitions in everyday life, and she felt more in control of her life.

Just over a decade since the first publication of the preliminary effectiveness of AVATAR therapy (Leff et al. 2013), this innovative therapy has developed and consolidated as an evidence‐based targeted digital therapy for distressing voices. The efficacy results of the multi‐center RCT (Garety et al. 2021) and the exploration of mechanisms of change in a future analysis will guide further refinement of the AVATAR approach. Access to AVATAR therapy (as used in the present study, Craig et al. 2018 and Garety et al. 2021) still remains limited to clinical‐research contexts, which include AVATAR‐trained therapists and supervisors, with access to the digital platform (not yet commercialized or available outside research). However, AVATAR therapy has recently received a National Institute for Health and Care Excellence Early Value Assessment (NICE‐EVA) recommendation as a digital health technology for psychosis (NICE 2024), which can potentially accelerate its wider availability in the UK National Health Service (NHS). This ongoing implementation work is occurring alongside further development of AVATAR therapy. Different studies have now shown the potential transdiagnostic application of AVATAR therapy, for example, in eating disorders (Cardi et al. 2022; Thompson et al. 2023). There is also work soon to be commenced on innovation in AI‐powered virtual conversational agents capable of delivering avatar dialogs (Wellcome reference: 227721/Z/23/Z). AVATAR_VRSocial brings immersive VR as a form of augmentation of AVATAR therapy gains, allowing to transfer the tailored and individualized voice as a distressing hearing experience during social situations and aiming to help the person to deal better with their voices in daily situations. AVATAR_VRSocial is currently being tested in a feasibility clinical trial in our MHRTC in Germany (ISRCTN35980117).

## References

[jclp23785-bib-0001] Bell, I. H. , J. Nicholas , M. Alvarez‐Jimenez , A. Thompson , and L. Valmaggia . 2020. “Virtual Reality as a Clinical Tool in Mental Health Research and Practice.” Dialogues in Clinical Neuroscience 22, no. 2: 169–177. 10.31887/DCNS.2020.22.2/lvalmaggia. 32699517 PMC7366939

[jclp23785-bib-0002] Cardi, V. , T. Ward , V. Aya , C. Calissano , A. Thompson , and J. Treasure . 2022. “A Proof‐of‐Concept Study for the Use of a Computerised Avatar to Embody the Eating Disorder Voice in Anorexia Nervosa.” Eating and Weight Disorders ‐ Studies on Anorexia, Bulimia and Obesity 27, no. 8: 3499–3506. 10.1007/s40519-022-01487-3. PMC9803737 36272035

[jclp23785-bib-0003] Craig, T. K. , M. Rus‐Calafell , T. Ward , et al. 2018. “AVATAR Therapy for Auditory Verbal Hallucinations in People With Psychosis: A Single‐Blind, Randomised Controlled Trial.” Lancet Psychiatry 5, no. 1: 31–40. 10.1016/S2215-0366(17)30427-3. 29175276 PMC5746597

[jclp23785-bib-0004] Freeman, D. , S. Lambe , T. Kabir , et al. 2022. “Automated Virtual Reality Therapy to Treat Agoraphobic Avoidance and Distress in Patients With Psychosis Gamechange): A Multicentre, Parallel‐Group, Single‐Blind, Randomised, Controlled Trial in England With Mediation and Moderation Analyses.” Lancet Psychiatry 9, no. 5: 375–388. 10.1016/s2215-0366(22)00060-8. 35395204 PMC9010306

[jclp23785-bib-0005] Garety, P. , C. J. Edwards , T. Ward , et al. 2021. “Optimising AVATAR Therapy for People Who Hear Distressing Voices: Study Protocol for the AVATAR2 Multi‐Centre Randomised Controlled Trial.” Trials 22, no. 1: 366. 10.1186/s13063-021-05301-w. 34034792 PMC8145186

[jclp23785-bib-0019] Haddock, G. , J. McCarron , N. Tarrier , and E. B. Faragher . 1999. “Scales to Measure Dimensions of Hallucinations and Delusions: The Psychotic Symptom Rating Scales (PSYRATS).” Psychological Medicine 29, no. 4: 879–889. 10.1017/S0033291799008661. 10473315

[jclp23785-bib-0006] Hardy, A. , N. Keen , D. van den Berg , et al. 2024. “Trauma Therapies for Psychosis: A State‐of‐The‐Art Review.” Psychology and Psychotherapy: Theory, Research and Practice 97, no. 1: 74–90. 10.1111/papt.12499. 37795877

[jclp23785-bib-0007] Leff, J. , G. Williams , M. A. Huckvale , M. Arbuthnot , and A. P. Leff . 2013. “Computer‐Assisted Therapy for Medication‐Resistant Auditory Hallucinations: Proof‐of‐Concept Study.” British Journal of Psychiatry 202: 428–433. 10.1192/bjp.bp.112.124883. 23429202

[jclp23785-bib-0008] National Institute for Health and Care Excellence (NICE) . 2024. “Digital Health Technologies to Help Manage Symptoms of Psychosis and Prevent Relapse in Adults and Young People: Early Value Assessment.” https://www.nice.org.uk/guidance/hte17/chapter/1-Recommendations.

[jclp23785-bib-0009] O'Brien, C. , M. Rus‐Calafell , T. K. Craig , et al. 2021. “Relating Behaviours and Therapeutic Actions During AVATAR Therapy Dialogue: An Observational Study.” British Journal of Clinical Psychology 60, no. 4: 443–462. 10.1111/bjc.12296. 33949726 PMC12086748

[jclp23785-bib-0010] Percie du Sert, O. , S. Potvin , O. Lipp , et al. 2018. “Virtual Reality Therapy for Refractory Auditory Verbal Hallucinations in Schizophrenia: A Pilot Clinical Trial.” Schizophrenia Research 197: 176–181. 10.1016/j.schres.2018.02.031. 29486956

[jclp23785-bib-0011] Pot‐Kolder, R. M. C. A. , C. N. W. Geraets , W. Veling , et al. 2018. “Virtual‐Reality‐Based Cognitive Behavioural Therapy Versus Waiting List Control for Paranoid Ideation and Social Avoidance in Patients with Psychotic Disorders: A Single‐Blind Randomised Controlled Trial.” Lancet Psychiatry 5, no. 3: 217–226. 10.1016/S2215-0366(18)30053-1. 29429948

[jclp23785-bib-0020] Rosebrock, L. , S. Lambe , S. Mulhall , et al. 2022. “Understanding Agoraphobic Avoidance: The Development of the Oxford Cognitions and Defences Questionnaire (O‐CDQ).” Behavioural and Cognitive Psychotherapy 50, no. 3: 257–268. 10.1017/S1352465822000030. PMC9378026 35166196

[jclp23785-bib-0012] Rus‐Calafell, M. , N. Ehrbar , T. Ward , et al. 2022. “Participants' Experiences of Avatar Therapy for Distressing Voices: A Thematic Qualitative Evaluation.” BMC Psychiatry 22, no. 1: 356. 10.1186/s12888-022-04010-1. 35610590 PMC9129894

[jclp23785-bib-0013] Rus‐Calafell, M. , P. Garety , E. Sason , T. J. K. Craig , and L. R. Valmaggia . 2018. “Virtual Reality in the Assessment and Treatment of Psychosis: a Systematic Review of Its Utility, Acceptability and Effectiveness.” Psychological Medicine 48, no. 3: 362–391. 10.1017/s0033291717001945. 28735593

[jclp23785-bib-0014] Rus‐Calafell, M. , T. Ward , X. C. Zhang , C. J. Edwards , P. Garety , and T. Craig . 2020. “The Role of Sense of Voice Presence and Anxiety Reduction in AVATAR Therapy.” Journal of Clinical Medicine 9, no. 9: 2748. 10.3390/jcm9092748. 32854387 PMC7564300

[jclp23785-bib-0015] Sheaves, B. , L. Johns , E. Černis , L. Griffith , and D. Freeman . 2021. “The Challenges and Opportunities of Social Connection When Hearing Derogatory and Threatening Voices: A Thematic Analysis with Patients Experiencing Psychosis.” Psychology and Psychotherapy: Theory, Research and Practice 94, no. 2: 341–356. 10.1111/papt.12303. PMC8247012 33124757

[jclp23785-bib-0016] Steel, C. , J. Schnackenberg , H. Perry , E. Longden , E. Greenfield , and D. Corstens . 2019. “Making Sense of Voices: a Case Series.” Psychosis 11, no. 1: 3–15.

[jclp23785-bib-0017] Thompson, A. , C. Calissano , J. Treasure , et al. 2023. “A Case Series to Test the Acceptability, Feasibility and Preliminary Efficacy of AVATAR Therapy in Anorexia Nervosa.” Journal of Eating Disorders 11, no. 1: 181. 10.1186/s40337-023-00900-1. 37833732 PMC10571357

[jclp23785-bib-0018] Ward, T. , M. Rus‐Calafell , Z. Ramadhan , et al. 2020. “AVATAR Therapy for Distressing Voices: A Comprehensive Account of Therapeutic Targets.” Schizophrenia Bulletin 46: 1038–1044. 10.1093/schbul/sbaa061. 32372082 PMC7505185

[jclp23785-bib-0021] Woodward, T. S. , K. Jung , H. Hwang , et al. 2014. “Symptom Dimensions of the Psychotic Symptom Rating Scales in Psychosis: A Multisite Study.” Supplement, Schizophrenia Bulletin 40, no. Suppl 4: S265–S274. 10.1093/schbul/sbu014. 24936086 PMC4141314

